# Contamination of Bananas with Beauvericin and Fusaric Acid Produced by *Fusarium oxysporum* f. sp. *cubense*

**DOI:** 10.1371/journal.pone.0070226

**Published:** 2013-07-26

**Authors:** Chunyu Li, Cunwu Zuo, Guiming Deng, Ruibin Kuang, Qiaosong Yang, Chunhua Hu, Ou Sheng, Sheng Zhang, Lijun Ma, Yuerong Wei, Jing Yang, Siwen Liu, Manosh Kumar Biswas, Altus Viljoen, Ganjun Yi

**Affiliations:** 1 Institution of Fruit Tree Research, Guangdong Academy of Agricultural Sciences, Guangzhou, Guangdong Province, China; 2 Key Laboratory of South Subtropical Fruit Biology and Genetic Resource Utilization, Ministry of Agriculture, Guangzhou, China; 3 The College of Life Science, South China Agricultural University, Guangzhou, China; 4 Plant Pathology, Plant Soil and Insect Sciences, The College of Nature Sciences, University of Massachusetts, Broad Institute, Cambridge, Massachusetts, United States of America; 5 Department of Plant Pathology, University of Stellenbosch, Private Bag X1, Matieland, South Africa; 6 Proteomics and Mass Spectrometry Core Facility, Cornell University, Ithaca, New York, United States of America; 7 Key Laboratory of Horticultural Plant Biology, Ministry of Education, Huazhong Agricultural University, Wuhan, Hubei, China; NIAID, United States of America

## Abstract

**Background:**

Fusarium wilt, caused by the fungal pathogen *Fusarium oxysporum* f. sp. *cubense* (*Foc*), is one of the most destructive diseases of banana. Toxins produced by *Foc* have been proposed to play an important role during the pathogenic process. The objectives of this study were to investigate the contamination of banana with toxins produced by *Foc*, and to elucidate their role in pathogenesis.

**Methodology/Principal Findings:**

Twenty isolates of *Foc* representing races 1 and 4 were isolated from diseased bananas in five Chinese provinces. Two toxins were consistently associated with *Foc*, fusaric acid (FA) and beauvericin (BEA). Cytotoxicity of the two toxins on banana protoplast was determined using the Alamar Blue assay. The virulence of 20 *Foc* isolates was further tested by inoculating tissue culture banana plantlets, and the contents of toxins determined in banana roots, pseudostems and leaves. Virulence of *Foc* isolates correlated well with toxin deposition in the host plant. To determine the natural occurrence of the two toxins in banana plants with Fusarium wilt symptoms, samples were collected before harvest from the pseudostems, fruit and leaves from 10 Pisang Awak ‘Guangfen #1’ and 10 Cavendish ‘Brazilian’ plants. Fusaric acid and BEA were detected in all the tissues, including the fruits.

**Conclusions/Signficance:**

The current study provides the first investigation of toxins produced by *Foc* in banana. The toxins produced by *Foc*, and their levels of contamination of banana fruits, however, were too low to be of concern to human and animal health. Rather, these toxins appear to contribute to the pathogenicity of the fungus during infection of banana plants.

## Introduction

The genus *Fusarium* comprises a large number of plant-associated fungal species with the potential to cause economic damage and reduce the quality of agricultural crops. These include wilt pathogens such as *F. oxysporum* and *F. solani*, both which cause the eventual demise of their plant hosts, and mycotoxin-producers such as *F. graminearum* and *F. verticillioides*, which contaminate food and feed with secondary metabolites (mycotoxins) harmful to humans and animals. Some toxins produced by *Fusarium* species are important for the establishment of the fungus in host tissue, and are known as phytotoxins. Numerous mycotoxins and phytotoxins produced by *Fusarium* species have now been described, along with their potential to cause disease to plants and animals [1].

One of the most economically important *Fusarium* species is *F. oxysporum*, a soil-borne saprophyte with specificity to more than 150 different plant hosts [2]. Most of the pathogenic members of the species attack a single or a couple of hosts, which allow them to be grouped into *formae speciales*. Pathogenic *F. oxysporum* isolates infect their hosts through the roots, invade the xylem vessels and eventually cause a lethal wilting of the infected plant. Wilting is the result of the restrictive movement of water in the vascular bundles [3], but part of the pathogenesis and invasion of plants by *F. oxysporum* can be contributed to toxic metabolites produced by the fungus. Toxins produced by *F. oxysporum* include beauvericin (BEA), the enniatines, fusaric acid (FA), moniliformin, naphthazarins and sambutoxin [1]. Important mycotoxins produced by other *Fusarium* species with health consequences to humans and animals include fumonisins, the trichothecenes (T2-toxin, nivalenol and deoxynivalenol) and zearalenone.

Phytotoxins such as FA cause pathological changes in the plant, including browning of vascular cells and plant necrosis [4]. Fusaric acid is a non-specific phytotoxin that is produced by many fungal pathogens to cause diseases in plants [5]. The toxin induces cell membrane early super polarization [4], suppresses H^+^ pumping and causes K^+^ leaking. It also suppresses mitochondrial oxygen absorption and causes malic acid oxidation [6]. Fusaric acid is capable of conjugating with Cu, Co, Fe and Zn, forming chelates which make these minerals unavailable to plants [7]. It further inhibits the activities of plant defensive enzymes and leads to a reduction in plant cell viability [8]. These processes quickly provoke other physiological reactions such as the production of reactive oxygen species [9], changes in membrane permeability [10], and changes in membrane potential [6].

Beauvericin is produced by the entomopathogenic fungus *Beauveria bassiana*, which attacks insects [11], and by several species from the *Fusarium fujikuroi* species complex [1], [12]. The mycotoxin has first been found in *F. oxysporum* from corn [13], and has since been described in several *formae speciales* of *F. oxysporum* [14]. Using an artificial membrane as a model to study the mode of action of BEA, Lemmens-Gruber *et al*. [15] showed that trace amounts of BEA formed a channel in the biofilm. Pavlovkin et al. [16] demonstrated that BEA treatment creates a rapid membrane electropotential in the corn root outer cortex cells, followed by a significant depolarization, and Hyuk-Hwan Song also proved BEA exhibit phytotoxicity on potato [17]. However, a study by Moretti et al. (2002) involving *F. oxysporum f. sp. melonis* demonstrated that BEA is neither related to its pathogenicity, nor to the differential specificity of its races [18].

*Fusarium oxysporum* f. sp. *cubense* (*Foc*), the causal agent of banana Fusarium wilt, is considered one of the most important pathogens of banana (*Musa* spp.) plants [4]. Banana is considered the fourth most important staple food crop and fruit in the world [19], and the loss of plantations due to *Foc* may result in loss of food and income to millions of people. Furthermore, chlamydospores produced by the fungus will survive in infested soils for decades, making the continued production of bananas difficult [20]. Depending on the *Foc* race present in banana fields, resistant varieties can be planted to continue production [21]. Gros Michel and Pisang Awak bananas are susceptible to *Foc* race 1, but Cavendish bananas are resistant to this race and *Foc* race 2. Cavendish bananas, however, are susceptible to *Foc* race 4 isolates, which also attack all other banana varieties susceptible to *Foc* races 1 and 2 [20].

Matsumoto et al. [22] reported that banana clones resistant to *Foc* can be differentiated from susceptible ones by treating the apical meristem with a fungal crude filtrate. Xu et al. [23] proposed that FA was the main component of the crude toxin produced by *Foc* which caused Fusarium wilt of banana. However, Morpurgo et al. [24] could not establish a clear link between the *in vivo* and *in vitro* behavior of banana shoot tip cultures treated with FA and fungal crude filtrates, and those inoculated with *Foc*. Their results, therefore, suggested that the use of crude filtrate and the non-host specific toxin FA in a screening program for selecting resistant banana genotypes is not necessarily accurate. More recently, however, BEA was associated with *Foc* isolates collected from apple banana in the Americas [25].

Due to the devastating effect of *Foc* to banana production, most studies on the pathogen have focused on its diversity, geographical distribution, symptomology, epidemiology and management [2], [26]-[31]. Little attention, however, has so far been paid to phytotoxic and food safety issues concerning *Foc*-infected banana. The first objective of this study, thus, was to confirm the range of toxins produced by *Foc*. A second objective was to correlate toxin production in banana plants to fungal virulence. Such information may provide new information to elucidate pathogenicity of *Foc* to banana plants. The final objective was to determine the concentration of *Foc* toxins in banana plants. This may signify whether infection of banana plants with *Foc* presents a food safety risk.

## Materials and Methods

### Fungal Isolates Used

Forty *Foc* isolates obtained from diseased banana pseudostems were used for studies on secondary metabolite production and no specific permits were required for the described field studies. These isolates were all collected from Cavendish cv ‘Brazilian’ (AAA) and Pisang Awak cv ‘Guangfen #1’ (ABB) banana plants with Fusarium wilt symptoms in Guangdong, Hainan, Fujian, Yunnan and Guangxi Provinces in China. The fungal cultures were all identified as *F. oxysporum* based on cultural appearance, the morphology of macroconidia, the production of microconidia in false head on monophialides, and the presence of single or pairs of chlamydospores. They were confirmed as the banana Fusarium wilt pathogen by completing pathogenicity tests, and divided into vegetative compatibility groups (VCGs) according to heterokaryon formation with known *Foc* VCG testers (Chunyu Li, unpublished data). The *Foc* isolates included seven *Foc* race 1 isolates and 33 *Foc* race 4 isolates (Chunyu Li, unpublished data) (Tables 1 and 2). All the isolates are maintained at the Agricultural Culture Collection of China (ACCC).

**Table 1:** Origin, race, and *in vitro* toxin production of isolates of *Fusarium oxysporum* f. sp. *cubense.*

Isolates^a^	Origin	Race	BEA(µg/g)^bc^	FA(µg/g)^bc^
ACCC 37969	GD-Panyu	1	10.5±4.0^DEF^	110±3.7^BC^
ACCC 37972	GD-Panyu	4	22.1±1.2^C^	75.2±9.9^D^
ACCC 37967	GD-Zhongshan	1	2.07±1.0^FG^	6.83±2.1^EF^
ACCC 37968	GD-Zhongshan	4	24.7±2.5^C^	165±2.4^A^
ACCC 37965	GD-Dongguan	4	2.29±1.6^EFG^	2.85±2.0^EF^
ACCC 37966	GD-Zhanjiang	4	15.3±3.6^CD^	17.66±3.56^EF^
ACCC 37974	GD-Zhanjiang	1	10.6±4.0^DEF^	16.0±2.3^EF^
ACCC 37980	HN-Chenghai	4	15.7±4.7^CD^	99.3±14.7^BCD^
ACCC 37981	HN-Haikou	4	4.22±1.6^EFG^	4.28±2.0^EF^
ACCC 37982	HN-Ledong	4	23.0±8.9^C^	123.67±1.59^B^
ACCC 37983	HN-Chengmai	4	39.9±8.5^B^	181±44.9^A^
ACCC 37960	FJ-Zhangzhou	1	15.6±4.0^CD^	26.6±5.3^E^
ACCC 37961	FJ-Zhangzhou	4	11.8±4.1^DE^	12.7±3.0^EF^
ACCC 37962	FJ-Zhangzhou	4	3.83±1.3^EFG^	4.65±2.1^EF^
ACCC 37963	FJ-Zhangzhou	4	62.7±12.5^A^	96.7±21^CD^
ACCC 37994	YN-Jinghong	4	16.7±6.6^CD^	24.5±6.4^EF^
ACCC 37995	YN-Hekou	4	2.29±1.1^EFG^	7.23±1.5^EF^
ACCC 37978	GX-Wuming	4	7.01±2.4^DEFG^	15.2±3.7^EF^
ACCC 37979	GX-Nanning	4	4.72±1.0^EFG^	91.2±18^CD^
ACCC 37976	GX-Fubei	4	22.8±1.6^C^	170±26^A^
Control			0	0

a The *Fusarium* isolates were maintained at Agricultural Culture Collection of China (ACCC).

b Detection limits for beauvericin (BEA) and fusaric acid (FA) were 1.8 and 4.8ng/g respectively.

c The data were the means of three replications. Mean values in the same column followed by the different letter are significantly different by Fisher’s protected least significant difference test (P<0.05).

**Table 2:** In Vitro toxin production of isolates of *Fusarium oxysporum* f. sp. *cubense*.

Isolates^a^	Origin	Host^b^	Race	BEA (µg/g)^c^	FA (µg/g)^c^
ACCC37985	Panyu	Br	4	3.15±0.62^GHIJK^	4.2±1.3^ABCD^
ACCC37986	Panyu	Br	4	55.4±2.1^B^	4.25±1.7^ABCD^
ACCC37987	Panyu	Br	4	11.5±1.3^C^	2.25±1.8^CD^
ACCC37988	Panyu	Br	4	1.66±0.55^JKL^	1.35±1.1^CD^
ACCC37989	Panyu	Br	4	4.25±1.2^FGHI^	3.24±2.0^BCD^
ACCC37950	Dongguan	Br	4	6.45±0.91^DEF^	1.25±1.1^CD^
ACCC37951	Dongguan	Br	4	4.32±0.75^FGHI^	3.56±2.8^BCD^
ACCC37952	Dongguan	Br	4	61.9±4.1^A^	5.65±3.7^ABC^
ACCC37953	Dongguan	Br	4	7.36±0.56^DE^	8.54±4.8^A^
ACCC37954	Dongguan	Br	4	2.65±0.28^HIJK^	1.34±0.3^CD^
ACCC37990	Panyu	GF	1	1.25±0.23^KL^	1.22±0.8^CD^
ACCC37991	Panyu	GF	1	5.26±1.6^EFG^	5.64±4.1^ABC^
ACCC37973	Panyu	GF	4	8.12±1.5^D^	8.55±6.5^A^
ACCC37992	Panyu	GF	4	3.77±0.58^GHIJ^	4.15±3.1^ABCD^
ACCC37993	Panyu	GF	4	2.36±0.27^IJK^	2.22±1.5^CD^
ACCC37955	Dongguan	GF	1	3.66±0.31^GHIJ^	4.25±2.6^ABCD^
ACCC37956	Dongguan	GF	4	6.22±2.0^DEF^	5.65±4.6^ABCD^
ACCC37957	Dongguan	GF	4	8.25±2.1^D^	7.65±6.1^AB^
ACCC37958	Dongguan	GF	4	4.85±0.52^FGH^	3.95±3.2^ABCD^
ACCC37959	Dongguan	GF	4	4.66±1.3^FGH^	3.96±1.6^ABCD^
Control1				0	**0**
Control2				0	**0**

a The *Foc* isolates were maintained at Agricultural Culture Collection of China (ACCC) and Control1 and Control2 were inoculated with sterile deionised water.

b Banana plant host: Br, Brazilian; GF, Guangfen #1”.

c Detection limits for BEA and FA were 6.0 and 4.8ng/g respectively, and the data were the means of three replications. Mean values in the same column followed by the different letter are significantly different by Fisher’s protected least significant difference test (P<0.05).

### Mycotoxin Analyses

*Fusarium oxysporum* f. sp. *cubense* isolates were inoculated onto potato dextrose agar (PDA) plates and cultured in the dark at 28°C. After 15 days, the mycelia were harvested, sonicated and the metabolites extracted with methanol. The extracts were analyzed by high performance liquid chromatography - tandem mass spectrometry (HPLC-ESI-MS; LCQDECA XP PLUS; Thermo Finnigan, Thermo Corp., Rockford, IL, USA) [32], using methanol and water (50∶50, v:v) as the mobile phase. The electrospray ionization source operating voltage was 3.01 kv, with the operating temperature at 250.3°C and an operating pressure of 0.1 bar. Mycotoxins used as standards included aflatoxin B1, B2, G1, G2 and M1 (AFB1, AFB2, AFG1, AFG2 and AFM1), ochratoxin A (OTA), T-2 toxin, zearalenone (ZON), sterigmatocystin (SMC) and citrinin (CTN), all purchased from Alexisa (San Diego, CA, USA); 3-Acetyldeoxynivalenol (3-ADON), fusarenone X (FX), deoxynivalenol (DON), BEA, FA, nivalenol (NIV), fumonisin (F) B1 (FB1) and B2 (FB2), enniatine A1 and B1, ZON, benzoic acid lactone, internal standard, IS) and verruculogen (VCG) supplied by Sigma–Aldrich (St. Louis, MO, USA); and 15-acetyldeoxynivalenol (15-ADON) and HT-2 toxin purchased from Biopure (Tulln, Austria). These toxins were selected because of their occurrence in *F. oxysporum* and other phytopathogenic *Fusarium* species [1].

### Toxicity of *Foc* metabolites to Banana

The effect of FA and BEA produced by *Foc* to banana was first determined on cell protoplast. Banana protoplasts were obtained from embryogenic cell suspensions (ECS) [33] of the cultivar ‘Dongguan Dajiao’ (ABB). The viable protoplasts were isolated from the ECS at a yield of 1.2×10^7^ protoplasts/ml packed cell volume (PCV), according to the method of Xiao et al. [34]. The protoplasts were then suspended in 20 ml of pre-plasmolysis buffer (25 mM Tris–Mes pH 7.4) containing 0.6 M sorbitol, 0.5% BSA and 0.5% CaCl_2_.

Cytotoxicity of FA and BEA to banana protoplasts was tested using the Alamar Blue assay according to manufacturers’ recommendations (Biotium, Cat: 30025-1). The FA and BEA were first dissolved in 0.5% methanol to prepare a stock solution of 1 mM, which was then serially diluted to concentrations of 200, 50, 10 and 1 µM using pre-plasmolysis buffer. The control treatment included equal amounts of methanol and pre-plasmolysis buffer. The reactions were performed in 96-well plates, each well containing 2×10^4^ cells in 100 µl of test solution. The results were calculated with a multifunctional microplate reader after 6, 12, 24, 36, 48 and 72 hours. Damage to cells was also observed under a laser scanning confocal microscope (Leica, Mannheim, Germany) with a wavelength of 488 nm. Each reaction was repeated three times, and the statistical software used was SAS 8.0 (P<0.05).

The effect of FA and BEA was further tested on banana pseudostems and tissue culture plantlets. The toxins were dissolved in 0.5% methanol to prepare a stock solution of 1 mM, which was then serially diluted to obtain concentrations of 500, 300, 200 and 100 µM. For treatment, the lower pseudostems of 6-week-old Cavendish cv ‘Brazilian’ (AAA) banana tissue culture plantlets were surface-disinfected with ethanol and dried. The base of each pseudostem was treated with 200 µl of FA and BEA solutions at different concentrations. For controls, the pseudostems were treated with the same amount of distilled water and 0.5% methanol, respectively. Treated pseudostems were placed on a layer of Whatman filter paper which was soaked in sterilized water in a Petri dish (90-cm diameter). The lid of the Petri dish was wrapped with parafilm to maintain the moisture. In addition, ‘Brazilian’ banana tissue-culture plantlets were transplanted into pre-plasmolysis buffer in test tubes containing 1, 5, 10, 20, 50, 100, 200, 300 and 500 µM FA and BEA. Both the Petri dishes and the treated pseudostems or tissue culture plantlets were incubated in a growth chamber with a photoperiod of 12 hours of light per day for 10 days at 3000 lux at 25°C. Each treatment was repeated three times, and statistically compared using SAS 8.0 software.

### Correlating Virulence and Toxin Production of *Foc* Isolates

Healthy tissue-culture plantlets of ‘Brazilian’ (AAA) and Pisang Awak cv ‘Guangfen #1’ (ABB) bananas at the 5- to 6-leaf stage were used for pathogenicity studies. *Foc* race 1 isolates were inoculated onto the ‘GuangFen #1’ plantlets, while *Foc* race 4 isolates were inoculated onto ‘Brazilian’ plantlets. The planting medium (six parts vermiculite, two parts peat, and one part coconut coir) was first sterilized in an autoclave, and the plants was inoculated with a spore suspension at a concentration of 10^5^ conidia/g soil, as described by Sun and Su [35]. Thirty plantlets were inoculated with each fungal isolate, and divided into three groups of ten plants each that were arranged in a randomized block design. All the inoculated plants were kept in a greenhouse, with the temperature ranging from 21–35°C, the humidity set at 40%, and the soil moisture maintained at about 60%. Disease symptoms that developed on banana plantlets were rated 30 days after inoculation. A disease index for individual plantlets was determined [36], and an integrated disease index for each isolate calculated using the following formula: Integrated disease index = Σ disease index×number of diseased plants. The roots, lowest leaves and lower pseudostem (3 cm from soil surface) of plantlets subjected to the different treatments were also collected for extraction of toxins.

### Distribution of BEA and FA in Mature Banana Plants

Ten ‘Guangfen #1’ and ten ‘Brazilian’ banana plants that developed Fusarium wilt symptoms under field conditions were selected for analyzing *in planta* mycotoxin contamination. The fruit, pseudostem and leaves of each plant were sampled at the fruit ripening stage, FA and BEA extracted with methanol [37], [38], and fungi contaminating isolated from diseased pseudostems and identified. For toxin extraction, 20 g of banana tissue was ground into a fine powder in liquid nitrogen, and the toxins extracted by adding 200 ml methanol for 24 hours. This process was repeated twice. The extracts were condensed to 1 ml and filtered through an Acrodisk filter (pore size 0.22 µm) (Millipore, Jonezawa, Japan) before LC/MS/MS analysis.

### LC/MS/MS Analysis of FA and BEA

The content of FA and BEA was determined using LC/MS/MS analysis. For FA analysis, mobile phase A consisted of acetonitrile:water:formic acid = 5∶95:0.1 (v:v:v), and mobile phase B of acetonitrile:water:formic acid = 95∶5:0.1 (v:v:v), with a flow rate of 0.2 ml/min using a gradient elution method. The column temperature was set at room temperature. Mass spectrometry employed an electron spray source in the positive ion mode 5.5 kv, and the scan mode was set up as selected reaction monitoring (SRM) method. During quantitative analysis, the ion reaction for FA was 180.1/134.2, for phenacetin it was 180.1/138.3 with the following parameters: scan time 0.1 s, heating temperature 350^o^C, atomization gas (N_2_) pressure 0.6 Mpa, auxiliary gas (N_2_) pressure 0.6 MPa; curtain gas (N_2_) pressure 0.4 MPa, focusing voltage (FP) 375 ev; collision gas (N_2_) pressure 0.4 MPa; NEB 8, CUR 10, and CAD 8. In order to quantify FA in the samples, the standard curve was obtained with solutions at 0.5, 1, 2, 5, 10, 20, 50, 100 µg/ml. If the concentration of FA in the sample was higher or the estimated curve was larger than the linearity range of the standard curve, the concentration of the sample was diluted with methanol.

For BEA analysis, LC/MS/MS was performed using a Finnigan LCQ Deca XP system (Thermo-Finnigan, San Jose, CA, USA) comprising a Surveyor liquid chromatograph, a Surveyor autosampler and an LCQ Deca XP quadrupole ion trap mass spectrometer equipped with an ESI source. The HPLC separation was performed using an ODS HYPERSIL column (2.1 mm id×150 mm, 3 µm). Elution was carried out using a mobile phase comprising water (15%) and methanol (85%) at a flow rate of 0.15 ml/min. LC/MS/MS conditions were as follows: spray voltage 3 kv, sheath gas flow 15 (arbitrary units). Relative collision energy was set at 60%. The BEA standard curve was obtained with solutions at 6, 15, 30, 60, 150, 300, 600 ng/ml. All analyses were performed in triplicates, and the mean values are reported here. Results were expressed at µg g^−1^ dry weight.

## Results

### Mycotoxin Analyses

**Figure 1:**
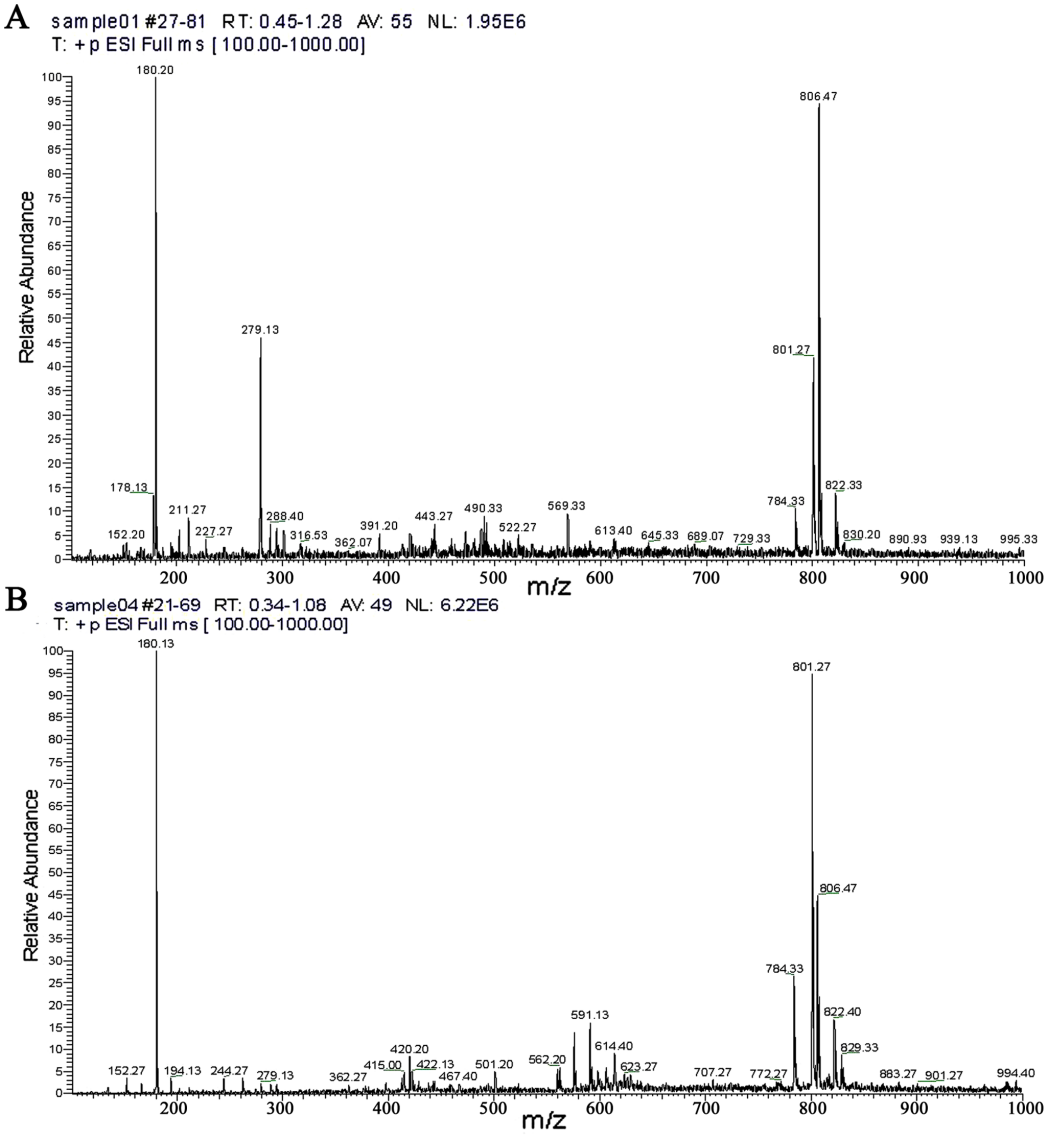
HPLC-ESI-MS map of the secondary metabolites from *Fusarium oxysporum* f.sp. *Cubense.* A: [M+H]^+^ map of *Foc* Race 1; B. [M+H]^+^ map of *Foc* Race 4.

Of all the mycotoxin tested for, only two, BEA and FA, were detected in the 20 *Foc* isolates selected (Figure 1). When the electrospray ionization mass spectra of the 20 *Foc* isolates were compared they showed several common characteristic peaks, such as m/z 180.13, m/z 784.33, m/z 801.27 and m/z 806.47 (Figure 1A and 1B). Signal m/z 180.13 represented FA production by *Foc*, while signals m/z 784.33, m/z 801.27 and m/z 806.47 were believed to represent BEA with different combinations of H, NH_3_ and Na groups.

### Toxicity of *Foc* Metabolites to Banana

**Figure 2:**
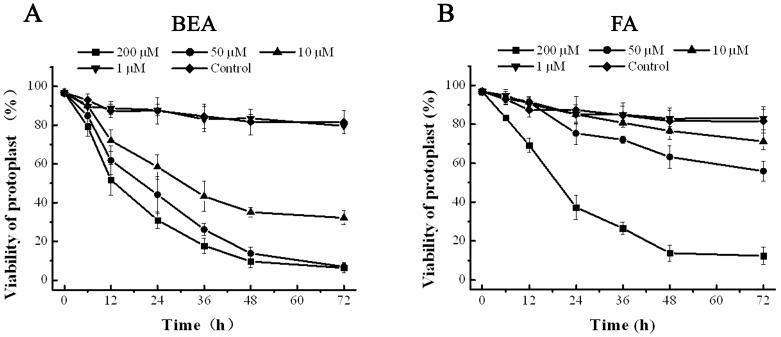
Cytotoxicity of FA and BEA to banana protoplasts using the Alamar Blue assay.

**Figure 3:**
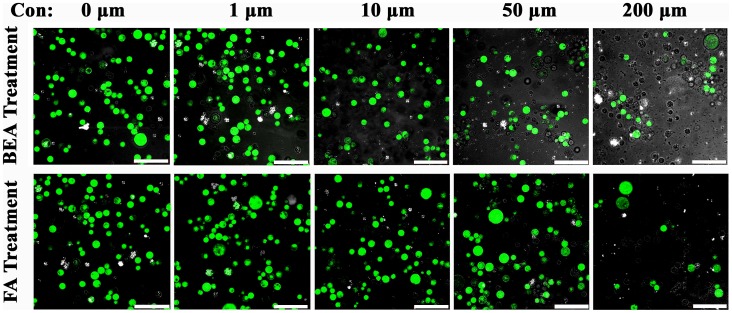
Toxicity analysis of different concentration of BEA and FA on protoplast at 72 **h.** The scale bar is 100 µm.

Fusaric acid and BEA were both toxic to banana protoplast (Fig. 2 and 3). At a concentration of 200 µM, FA reduced the viability of protoplasts to less than 20% after 48 hours. The phytotoxin proved less toxic at concentrations of 50 and 10 µM, killing an average of only 40 and 20% of protoplasts after 72 hours, respectively. BEA killed protoplasts more rapidly, and their viability was reduced to less than 20% after 48 hours of treatment at concentrations of 50 and 200 µM (Fig. 2). At 10 µM, it also reduced protoplast viability to less than 40% at the same time. No significant toxic effect was evoked by either FA or BEA at a concentration of 1 µM after 72 hours (Fig. 3).

**Figure 4:**
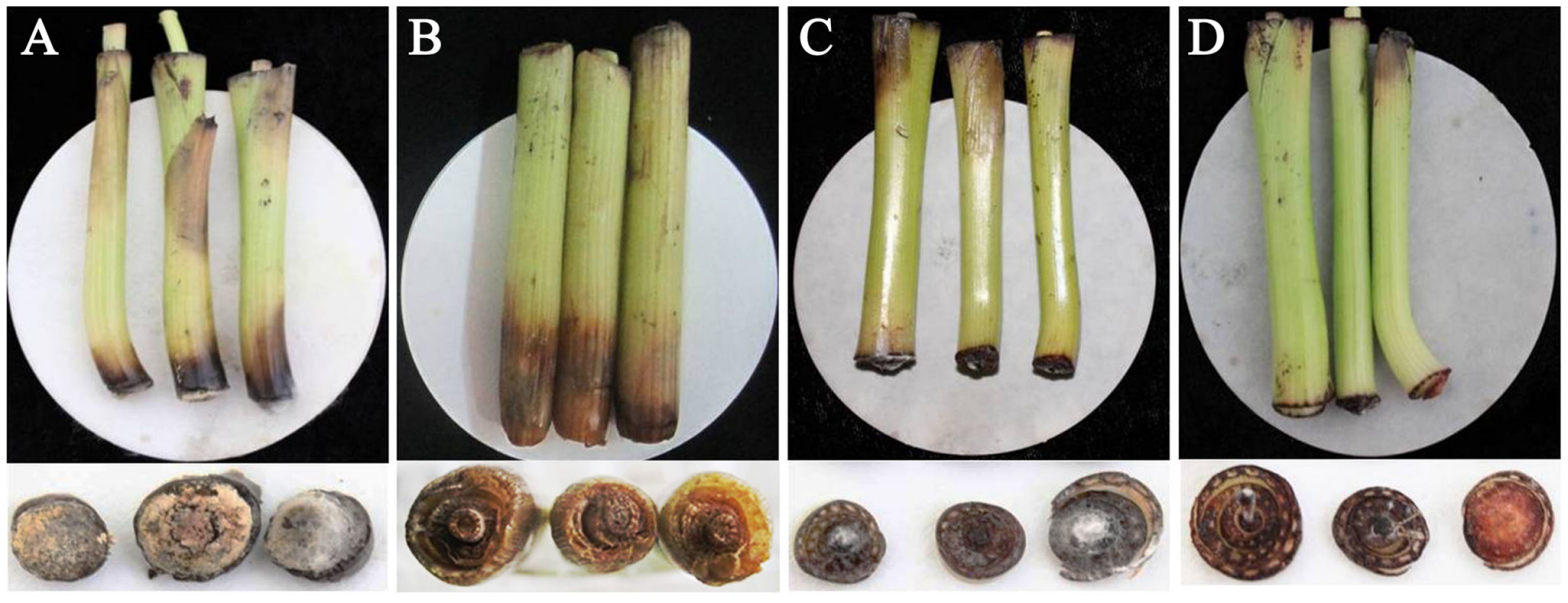
Treatment results of BEA and FA on banana pseudostem. A. the treatment of 1.0 mM BEA; B. the treatment of solvent (0.5% methanol); C. the treatment of water; D. the treatment of 1.0 mM FA.

Banana pseudostems treated with FA and BEA became eroded *in vitro*, with symptoms becoming more evident as the concentration of the phytotoxins was increased (Fig. 4; Table 3). Beauvericin proved to be significantly more toxic than FA, and resulted in more pronounced symptoms. The bottom surface of pseudostems treated with BEA became white and dried up, while the upper tissue was black and eroded. Pseudostems treated with FA became corroded and water-soaked. The solvent- and water-treated control pseudostems only developed a natural browning over time (Figure 4).

**Table 3:** Banana pseudostem eroded percentage caused by different concentration of BEA and FA.

	Control 1^a^	Control 2^a^	100 µM^b^	200 µM^b^	300 µM^b^	500 µM^b^	1000 µM^b^
BEA	0.98%±0.3	2.63%±0.4	3.4%±0.5^A^	4.1%±0.7^A^	4.5%±1.1^A^	5.2%±0.8^A^	11.9%±0.7^A^
FA	0.98%±0.3	2.63%±0.4	3.3%±0.7^A^	3.7%±0.4^B^	4.1%±1.2^B^	4.7%±0.9^B^	9.2%±0.8^B^

a Control 1 =  water; Control 2 = 0.5% methanol.

b The data were the means of three replications. Mean values in the same column followed by the different letter are significantly different by Fisher’s protected least significant difference test (P<0.05).

**Figure 5:**
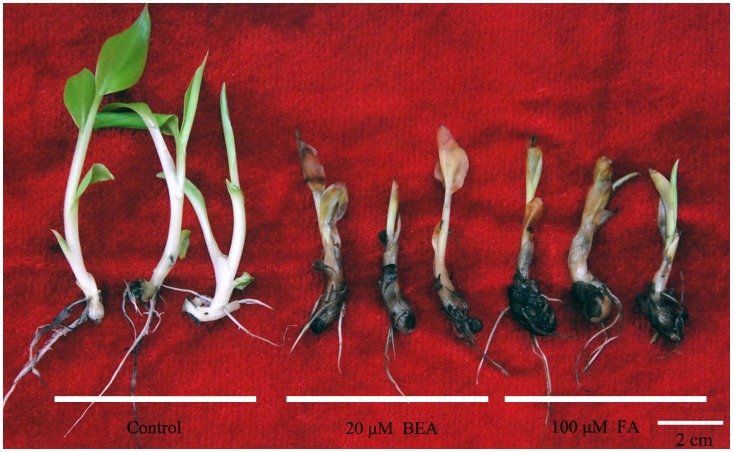
Toxicity analysis of BEA and FA on banana plantlets.

Fusaric acid and beauvericin caused banana plantlets to wilt, deteriorate and die *in vitro* with symptoms becoming more severe as concentrations were increased. At 1 µM, the leaves of plantlets turned yellow and became wilted, and the roots became slightly brown. When treated with BEA at a concentration of 50 µM, the parts of the plantlets were immersed in the buffer were softened in the solution. The same happened when plantlets were treated with 200 µM FA. Also, Beauvericin applied at 20 µM and FA applied at 100 µM produced the same symptoms, except that BEA resulted in dried leaf edges and FA not (Figure 5).

### *In vitro* Production of Mycotoxins

The *Foc* race 1 and race 4 isolates produced BEA on rice medium at concentration ranging from 2.07±1.02 to 62.65±12.54 µg/g, and FA at concentration from 2.85±2.01 to 180.63±44.68 µg/g. Nine isolates produced significantly more FA than BEA, including ACCC37969, 37972, 37968, 37980, 37982, 37983, 37979, 37976 and 37963 (Table 1).

### Correlating Virulence and Toxin Production by *Foc* Isolates

*Fusarium oxysporum* f. sp. *cubense* was divided into two groups: pathogenic and attenuated isolates. The pathogenic isolates included ACCC37969, 37972, 37968, 37980, 37982, 37983, 37979, 37976 and 37963, with an integrated disease index of more than 80. These isolates infected 100% of the inoculated plants, of which more than 50% showed a disease index of 4. The rest of the isolates were attenuated, with an integrated disease index of between 31 and 50. Ten percent of the inoculated plants developed no disease, while 70% of plants showed a disease index between 1 and 2. Only 20% reached a disease index of 4 [30]. *Fusarium oxysporum* f. sp. *cubense* isolates from the same or different geographic regions, even when they belong to the same race, showed different virulence levels (Table 1 and 4).

**Table 4:** Pathogenicity and amounts of toxins in banana root (R), leaves (L) and pseudostem (P) by isolates of *Fusarium oxysporum* f. sp. *cubense.*

Isolates^a^	IDI^b^	Production of BEA(ng/g)^c^			Production of FA (ng/g)^c^		
		R	L	P	R	L	P
37969^d^	80	9.85±1.2^C^	1.66±0.3^F^	10.3±1.3^C^	1110±140^D^	311±7.0^E^	1300±120^BC^
37972^e^	85	9.48±1.6^C^	2.97±0.2^D^	12.7±1.3^ABC^	1270±130^BCD^	331±35^E^	1390±160^BC^
37967^d^	31	1.32±0.14^D^	0.22±0.04^HI^	0.97±0.17^D^	114±39.0^EFG^	71.0±26.0^FGH^	145.0±17.0^DE^
37968^e^	105	11. 9±2.0^BC^	2.11±0.24^E^	12.50±2.4^ABC^	1500±330^AB^	435±61.0^CD^	1530±350^AB^
37965^e^	32	2.36±0.3^D^	0.64±0.11^G^	2.14±0.22^D^	234±44.0^EFG^	68.0±22.0^FGH^	278±26.0^DE^
37966^e^	45	2.03±0.31^D^	0.31±0.02^GHI^	2.52±0.35^D^	354±35.0^E^	115±25.0^F^	368±54.0^D^
37974^d^	47	2.25±0.26^D^	0.28±0.03^GHI^	2.15±0.34^D^	326±36.0^EF^	102±31.0^F^	346±34.0^D^
37980^e^	80	11.7±1.9^BC^	3.12±0.37^D^	11.4±2.4^BC^	1250±140^CD^	303±25.0^E^	1230±160^C^
37981^e^	41	2.41±0.26^D^	0.58±0.04^GH^	2.16±0.3^D^	263±31.0^EF^	98±12.0^F^	296±33.0^D^
37982^e^	102	13.4±2.8^AB^	3.24±0.33^D^	14.2±3.1^AB^	1300±260^BCD^	394±49.0^D^	1400±360^BC^
37983^e^	111	15.4±3.7^A^	4.99±0.41^A^	15.4±3.0^A^	1550±200^A^	462±47.0^BC^	1690±200^A^
37960^d^	45	2.09±0.24^D^	0.37±0.03^GHI^	2.35±0.38^D^	311±14.0^EF^	96±14.0^F^	324±15.0^D^
37961^e^	50	1.97±0.28^D^	0.27±0.01^GHI^	1.69±0.22^D^	286±28.0^EF^	90±8.0^FG^	295±15.0^D^
37962^e^	34	1.54±0.16^D^	0.25±0.01^GHI^	1.32±0.16^D^	114±11.0^EFG^	45±9.0^GHI^	126±17.0^DE^
37963^e^	105	13.9±3.2^AB^	3.66±0.41^C^	14.2±4.0^AB^	1480±160^ABC^	612±54^A^	1500±200^ABC^
37994^e^	36	1.31±0.11^D^	0.27±0.02^GHI^	1.64±0.19^D^	107±27^FG^	30.0±8.0^HI^	114±19^DE^
37995^e^	29	1.77±0.22^D^	0.26±0.03^GHI^	1.84±0.35^D^	123±25^EFG^	36±11^HI^	131±21^DE^
37978^e^	42	2.52±0.75^D^	0.47±0.03^GH^	2.28±0.36^D^	303±14^EF^	97±13^F^	325±20^D^
37979	96	13.0±2.8^AB^	3.82±0.43^C^	13.2±2.9^ABC^	1310±200^ABCD^	411±44^D^	1360±220^BC^
37976^e^	105	13.3±2.4^AB^	4.27±0.61^B^	13.7±3.1^AB^	1410±330^ABC^	458±59^CD^	1490±410^ABC^
Control^d^	0	0	0	0	0	0	0
Control^e^	0	0	0	0	0	0	0

a The *Foc* isolates were maintained at Agricultural Culture Collection of China (ACCC).

b IDI means Integrated Disease Index.

c Detection limits for BEA and FA were 6.0 and 4.8 ng/g respectively.

d Pisang Awak cv ‘GuangFen #1’ (ABB) was used as experimental plant.

e Cavendish cv ‘Brazilian’ (AAA) was used as experimental plant.

The data were the means of 3 replications. Mean values in the same column followed by the different letter are significantly different by Fisher’s protected least significant difference test (P<0.05).

Fusaric acid and BEA content in the root, the lower leaves and the pseudostems of the ‘Guangfen #1’ and ‘Brazilian’ banana showed that the more virulent the isolate, the higher the level of toxin produced in the host tissues (Table 4). Fusaric acid was produced substantially more than BEA, in some cases up to a 100 times. The roots and pseudostem contained more toxin than the leaves.

### Distribution of BEA and FA in Mature Banana Plants

The *Foc* isolates collected from diseased banana pseudostems produced BEA at concentrations of 1.25 to 61.85 µg/g, and FA at concentrations of 1.22 and 75.65 µg/g (Table 2). There were significant differences in the amount of BEA and FA contamination of plants from the same sampling site (Table 2).

In the field, Fusarium wilt-affected plants displayed various symptoms. The most seriously were wilt symptoms in leaves during the pre-flowering stage. These plants did not develop fruit, while less affected plants were able to develop buds, flowers and fruits. Only *Foc* were isolated from the pseudostems of diseased bananas. In fruits of ‘Brazilian’ and ‘Guangfen #1’ plants, FA was produced at concentrations of between 12.13 and 72.12 ng/g, and 13.35 and 46.57 ng/g, respectively (Table 5 and 6). Beauvericin concentration in these cultivars ranged from 5.66 to 40.53 pg/g, and from 9.56 to 30.12 pg/g, respectively. The pseudostem accumulated more FA than the banana fruit, with concentrations ranging from 1.32 to 10.36 µg/g and 7.03 to 36.62 µg/g in ‘Brazilian’ and ‘Guangfen #1’, respectively. Similarly, BEA production was more prolific, with concentrations between 1.25 and 5.66 ng/g in ‘Brazilian’ and from 4.57 to 16.51 ng/g in ‘Guangfen #1’ (Table 5 and 6). In the leaves, FA concentrations ranged between 65.62 and 298.78 ng/g in ‘Brazilian’, and from 24.65 to 332.56 ng/g in ‘Guangfen #1’. Beauvericin concentrations ranged from 0.21 to 2.36 ng/g and 0.23 to 6.65 ng/g in the two cultivars, respectively (Table 5 and 6).

**Table 5:** Isolated *Fusarium oxysporum* f. sp. *cubense* contaminanting and distribution of beauvericin (BEA) and fusaric acid (FA) toxins in *Musa* AAA Cavendish banana plant tissues.

Isolates^a^	Fruit		Pseudostem		Leaves	
	FA (ng/g)^b^	BEA (pg/g)^b^	FA (µg/g)	BEA (ng/g)	FA (ng/g)	BEA (ng/g)
37950	13.0	9.1	2.8	1.3	70.0	0.24
37951	72.0	41.0	8.8	3.3	210	1.0
37952	37.0	15.0	6.2	1.4	130	0.23
37953	10.0	5.7	8.3	3.6	67.0	0.36
37954	15.0	11.0	5.1	1.7	120	0.69
37985	12.0	9.6	6.4	1.7	140	0.45
37986	29.0	13.0	9.1	5.0	160	0.61
37987	52.0	25.0	10.0	5.7	30.0	2.4
37988	21.0	11.0	2.0	1.6	110	0.44
37989	27.0	12.0	1.3	1.4	66.0	0.21
Controll	ND	ND	ND	ND	ND	ND
Control2	ND	ND	ND	ND	ND	ND

Note:ND, Not Detected. Controll and Control2 were healthy Musa AAA Cavendish Brazilian from Panyu and Dongguan respectively.

a The *Fusarium* isolates were maintained at Agricultural Culture Collection of China (ACCC).

b Detection limits for BEA and FA were 6.0 and 4.8 ng/g respectively.

**Table 6:** Isolated *Fusarium oxysporum* f. sp. *cubense* contaminanting and distribution of beauvericin (BEA) and fusaric acid (FA) toxins in *Musa* ABB Pisang awak Guangfen #1 plant tissues.

Isolates a	Fruit		Pseudostem		Leaves	
	FA (ng/g)^b^	BEA (pg/g)^b^	FA (µg/g)	BEA (ng/g)	FA (ng/g)	BEA (ng/g)
37955	19.0	12.0	7.0	4.6	160.0	0.7
37956	13.4	9.6	8.6	5.1	56.0	0.6
37957	38.0	19.0	10.0	6.4	140.0	0.7
37958	39.0	30.0	21.0	12.0	320.0	1.6
37959	20.0	11.0	20.0	11.0	25.0	0.2
37990	26.0	16.0	17.0	9.3	54.0	0.3
37991	36.0	21.0	17.0	8.9	140.0	0.9
37992	47.0	29.0	19.0	11.0	250.0	1.5
37993	31.0	20.0	12.0	8.5	44.0	0.23
37973	37.0	15.0	37.0	17.0	330.0	6.7
Controll	ND	ND	ND	ND	ND	ND
Control2	ND	ND	ND	ND	ND	ND

Note: ND, not detected. Control1 and Control2 were healthy Musa ABB Pisang awak Guangfen #1 from Panyu and Dongguan respectively.

a The *Fusarium* isolates were maintained at Agricultural Culture Collection of China (ACCC).

b Detection limits for BEA and FA were 6.0 and 4.8 ng/g respectively.

## Discussion

Banana Fusarium wilt is widely believed to be caused by the colonization of the root, rhizome and pseudostem xylem vessels with *Foc*, and the subsequent blockage of the vascular system which eventually results in plant death [39]. The current study, however, demonstrated that FA and BEA, which are produced by *Foc* race 1 and 4 in diseased field bananas, also cause damage to the roots, pseudostem and leaves, thereby contributing to disease development. At cellular level, both FA and BEA can kill banana cell and protoplast, and at tissue levels, the symptoms include the rotting of roots and pseudostems, and the wilting of leaves of seedlings when applied at high concentrations. Both toxins, therefore, appear to play a much more important role in the pathogenic process than previously recognized.

A positive relationship has previously been reported between FA content and *F. oxysporum* isolates, for example, *F. oxysporum* f. sp. *niveum* (watermelon) [37], *F. oxysporum* f.sp. *tuberosi* (potato) [40], and *F. oxysporum* f. sp. *lycopersici* (tomato) [41]. In addition, BEA has been demonstrated to play an important role in the pathogenicity of Mycotoxigenic Fusarium avenaceum in wheat [17]. These findings were corroborated in the current study by demonstrating that the production of FA and BEA on rice medium and in banana plantlets could be positively correlated with the pathogenicity of 20 individual *Foc* isolates. The damage of banana protoplasts, root and pseudostems caused by *Foc* toxins increased cell membrane permeability and induced apoptosis [16], [42], thereby instigating wilt and plant death. Both BEA and FA have been widely associated with *F. oxysporum* [25], and do not distinguish between races and *formae speciales* of *F. oxysporum* [18]. This was also the case for *Foc* race 1 and race 4 isolates attacking Pisang Awak and Cavendish bananas, respectively.

*Fusarium oxysporum* f. sp. *cubense* can be isolated from all banana tissue, but is difficult to find in the upper stalk. It has also never been isolated from the fruit. In fact, both *Foc* race 1 and 4 isolates died when they were inoculated onto a medium made of banana pulp (Li, unpublished data). This situation appears to be somewhat different for melon, where BEA was detected inside the fruit of two cultivars after *F. oxysporum* f. sp. *melonis* was applied to the fruit surface [22]. The presence of FA and BEA in banana fruit rather suggests that they were translocated from the pseudostem and leaves where they were produced by *Foc*. The translocation of mycotoxins in plants is not an unusual event, and has been reported in corn and wheat [13], [17]. In banana, the fruit is separated from the pseudostem by a hard and dense stalk, which explains the low concentration of mycotoxins present in the fruit compared to that in the pseudostem.

Fusaric acid and BEA is not considered as mycotoxins with significant health consequences to humans and animals, and food and feed contaminated with these metabolites are therefore not regulated in any country. Still, both FA [43], [44] and BEA [15], [17], [45] have resulted in pathological disorders in experimental animals and human cell lines. For this reason, it is important to determine the extent to which humans and animals are exposed to toxins produced by *Foc* in banana plants. According to toxicological test in mice, the lethal concentration LC_50_ of FA is 80 mg/kg [44]. This suggests that a man of 50 kg will have to consume 10^5 ^kg bananas contaminated with FA at a concentration of 12.13–52.34 ng/g to be killed. Although the toxicity of FA may increase when combined with other mycotoxins [46], the BEA content in fruit of 5.66–40.53 pg/g was also low enough to be considered safe for human consumption [15], [43]. Furthermore, there has never been a report of intoxication of humans due to banana consumption. However, the issue might become prominent if the crop is utilized comprehensively as animal feed. Whereas banana fruit is considered safe for human consumptions, the mycotoxin content in infected pseudostems were up to a 1000-fold more, which could potentially be harmful to animals [47].

Mycotoxin production by *Foc* isolates proved to be more pronounced *in vitro* than *in planta*. This could be the result of sample collection, as toxins might be concentrated in some affected tissue and very low in other plant parts, which may result in their concentration being profoundly diluted in collected material. It is, however, more likely that the differences is attributed to very different environments; one in which the fungus is artificially cultured, and the other where it has to overcome plant structural and chemical barriers. More interesting is the significantly higher production of FA than BEA in plants. One can speculate the reason for this to be that (1) BEA is not stable, especially inside the banana plant where enzymes may catalyze its conversion to other compound, or that (2) the environment inside the plants is not favorable for BEA synthesis, and suppressed the expression of related genes. Yet, FA and BEA are simultaneously produced by *Foc* in banana, and it is important that their additive and synergistic effects in the pathogenic process be further investigated in future.
